# Does bronchial hyperresponsiveness predict a diagnosis of cough variant asthma in adults with chronic cough: a cohort study

**DOI:** 10.1186/s12931-021-01845-2

**Published:** 2021-09-23

**Authors:** Aleksandra Rybka-Fraczek, Marta Dabrowska, Elzbieta M. Grabczak, Katarzyna Bialek-Gosk, Karolina Klimowicz, Olga Truba, Rafal Krenke

**Affiliations:** Department of Internal Medicine, Pulmonary Diseases and Allergy, Medical University of Warsaw, ul. Banacha 1A, 02-097 Warsaw, Poland

**Keywords:** Asthma, Bronchial hyperresponsiveness, Cough

## Abstract

Bronchial hyperresponsiveness is a typical, but non-specific feature of cough variant asthma (CVA). This study aimed to determine whether bronchial hyperresponsiveness may be considered as a predictor of CVA in non-smoking adults with chronic cough (CC). The study included 55 patients with CC and bronchial hyperresponsiveness confirmed in the methacholine provocation test, in whom an anti-asthmatic, gradually intensified treatment was introduced. The diagnosis of CVA was established if the improvement in cough severity and cough-related quality of life in LCQ were noted.The study showed a high positive predictive value of bronchial hyperresponsiveness in this population. Cough severity and cough related quality of life were not related to the severity of bronchial hyperresponsiveness in CVA patients. A poor treatment outcome was related to a low baseline capsaicin threshold and the occurrence of gastroesophageal reflux-related symptoms. In conclusion, bronchial hyperresponsiveness could be considered as a predictor of cough variant asthma in non-smoking adults with CC.

To the Editor

Cough variant asthma (CVA) is a phenotype of asthma, characterized by bronchial hyperresponsiveness (BHR) and cough as a sole symptom [1, 2]. Despite some differences between the definitions of CVA, the presence of BHR and favorable response to anti-asthmatic treatment are two major criteria contributing to CVA diagnosis [3–5]. As CVA is a common cause of chronic cough (CC), the knowledge of its predictive factors is important for timely and effective diagnosis. Albeit BHR is a hallmark of CVA, its significance as a predictor of the response to classic anti-asthmatic therapy in adults with CC has not been evaluated so far. Hence, the above was the aim of our study.

## Materials and methods

### Study design, patients and definitions

This prospective, single-center, observational study (ClinicalTrials.gov NCT03363698) was performed in the Department of Internal Medicine, Pulmonary Diseases and Allergy of the Medical University of Warsaw between 2016 and 2020 and included non-smoking adults with CC suspected to have CVA. The study protocol was approved by the Institutional Review Board (KB/222/2016) and all patients signed informed consent.

Inclusion criteria were as follows: (1) age 18–85 years, (2) CC (lasting > 8 weeks), (3) no history of wheezing or dyspnea, (4) normal spirometry, (5) BHR in methacholine challenge [methacholine concentration causing 20% fall in FEV_1_ (PC_20_) below 16 mg/mL]. Exclusion criteria included: (1) acute respiratory tract infection within the previous 6 weeks, (2) treatment with inhaled corticosteroids (ICS) or long-acting β_2_-agonists (LABA) or leukotriene receptor antagonist (LTRA) or oral corticosteroids (OCS) or proton pump inhibitor (PPI) or antihistamine or intranasal corticosteroids within 4 weeks before enrollment, (3) abnormal chest radiograph, (4) active (within the last 12 months) smoking.

Upper airway cough syndrome (UACS) was defined as CC in a patient with chronic rhinitis or rhinosinusitis, diagnosed according to the respective guidelines [6–8]. Gastroesophageal reflux (GER) was diagnosed in patients who reported symptoms of GER or had a history of esophagitis revealed in upper gastrointestinal endoscopy or elevated number (> 73/24 h) of reflux episodes registered in 24-h pH-impedance monitoring [9].

### Pulmonary function and BHR measurement

All patients underwent pulmonary function testing, methacholine and capsaicin challenge as well as sputum induction before the onset of treatment. Spirometry was performed according to ERS guidelines (Lungtest 1000 MES, Krakow, Poland). FeNO was measured using Niox, Aerocrine, Solna, Sweden. Methacholine challenge was performed in a 2-min tidal breathing protocol (Lungtest 1000, MES Krakow, Poland), following the respective guidelines [10].

Capsaicin cough challenge was performed according to a previously recommended protocol using a single-breath method (Koko Digidoser, nSpire Health Inc., Longmont, USA) [9]. Cough reflex sensitivity was expressed as the lowest capsaicin concentrations evoking two (C2) and five (C5) coughs in the first 15 s after inhalation.

Sputum induction was performed as presented elsewhere [11].

### Treatment protocol and assessment of response

**Fig. 1:**
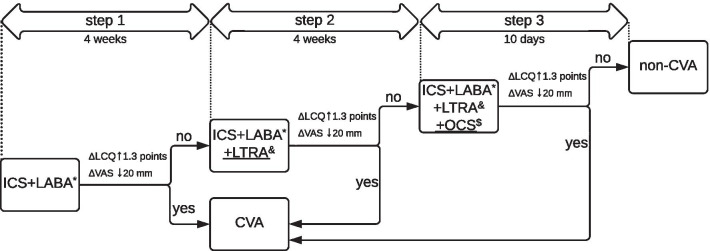
Stepwise approach to treatment of patients with chronic cough and bronchial hyperresponsiveness. The protocol of treatment was based on an add-on approach and included three consecutive steps. Therapy was initiated with a combination of a moderate dose of ICS + LABA (formoterol). If the improvement was reported (ΔLCQ + 1.3 points and ΔVAS − 20 mm from the baseline) after 4 weeks of treatment, the patient was diagnosed with CVA. However, if cough persisted, step 2 was initiated with add-on LTRA (montelukast 10 mg), with measurement of LCQ and VAS after the next 4 weeks of treatment. In case of the treatment failure, a short course (10 days) of OCS (0.5 mg/kg of prednisone) was introduced. The diagnosis of CVA was established if the improvement was noted after any of three steps *moderate dose of ICS according to GINA (pMDI: beclometasone dipropionate HFA, extrafine particle or ciclesonide or budesonide) in combination with formoterol 12-24 mcg daily; ^&^montelukast 10 mg daily; ^$^prednisone 0.5 mg/kg daily. CVA: cough variant asthma; GINA: The Global Initiative for Asthma; HFA: hydrofluoroalkane; ICS: inhaled corticosteroids; LABA: long-acting β2-agonists; ΔLCQ: change in Leicester cough questionnaire from the baseline; LTRA: leukotriene receptor antagonist; pMDI: pressurized metered-dose inhaler; OCS: oral corticosteroids; ΔVAS: change in cough severity from the baseline

Stepwise treatment protocol which included ICS, LABA, LTRA and OCS was applied in the study (see Fig. 1) [2, 5]. A significant response to treatment was defined as a decrease in cough severity from the baseline at least 20 mm [measured in visual analogue scale (VAS), range 0–100 mm] and improvement in quality of life (QoL) in Leicester Cough Questionnaire (LCQ, range 3–21) at least 1.3 points after one of three steps of therapy (Fig. 1) [9]. CVA was diagnosed if a patient met all inclusion criteria, did not meet any of the exclusion criteria and met the criteria of treatment response.

### Statistical analysis

Statistical analysis was performed using Statistica 13.3 software package (StatSoft, Tulsa, USA). Data were presented as median and interquartile range or numbers and percentages. Nonparametric tests: the Mann–Whitney U test, χ^2^ and Spearman rank’s correlation were used to study the inter-group differences and correlations. A *P* value lower than 0.05 was considered statistically significant.

## Results

### Study population and cough characteristics

Fifty-five patients meeting the inclusion criteria were selected from 250 adults with CC managed in the cough center. The study was completed by 49 patients (2 patients were lost to follow-up and 4 discontinued the treatment). The baseline patients’ characteristics are presented in Table 1. There was no correlation between PC_20_ and capsaicin cough provocation threshold.

**Table 1:** Patients’ characteristics and comparison between patients with bronchial hyperresponsiveness who responded (CVA patients) and not responded (non-CVA patients) to anti-asthmatic therapy

	All patients (N = 49)	Non-CVA (6; 12.2%)	CVA (43; 87.8%)	*P* value
Age (years)	60.0 (54.0–68.0)	54.5 (45.0–56.0)	61.0 (55.0–69.0)	0.0961
Gender (N female, %)	40 (81.6%)	5 (83.3%)	35 (81.4%)	0.6540
Cough duration (months)	48.0 (24.0–120.0)	84.0 (36.0–204.0)	48.0 (24.0–120.0)	0.5190
Smoking status (N ex-smokers, %)	12 (24.5%)	0 (0%)	31 (27.9%)	0.3260
Blood eosinophil count (cells/μL)	197.4 (128.3–303.7)	235.3 (157.5–405.0)	189.0 (120.7–284.2)	0.2578
BMI (kg/m^2^)	30.2 (25.8–33.1)	28.6 (26.8–31.4)	30.2 (24.7–33.2)	0.9878
FEV_1_ (% predicted)	86.0 (80.0–100.0)	92.5 (85.0–101.0)	86.0 (80.0–100.0)	0.4453
FeNO (ppb)	17.5 (12.2–27.0)	13.5 (10.9–24.5)	17.9 (13.7–27.1)	0.2891
PC_20_ (mg/mL)	2.0 (0.8–4.4)	2.2 (0.7–3.2)	2.0 (0.8–5.0)	0.6583
PC_20_ < 1 mg/mL (N patients, %)	14 (28.6%)	2 (33.3%)	12 (27.9%)	0.5590
PC_20_ < 4 mg/mL (N patients, %)	35 (71.4%)	5 (83.3%)	30 (69.8%)	0.4410
Sputum neutrophil percentage (%)	43.0 (34.0–52.0)	62.0 (49.0–62.0)	41.5 (31.5–51.5)	0.0291
Sputum eosinophil percentage (%)	1.0 (0.0–4.0)	0.0 (0.0–3.0)	1.0 (0.0–4.0)	0.5999
Atopy (N patients, %)	23 (46.7%)	2 (33.3%)	21 (48.8%)	0.7820
UACS (N patients, %)	38 (77.6%)	5 (83.3%)	33 (76.7%)	0.8730
GER (N patients, %)	27 (55.1%)	6 (100%)	21 (48.8%)	0.0183
Initial LCQ (points)	10.4 (8.4–13.1)	9.9 (9.1–10.9)	10.7 (8.3–13.8)	0.6148
Change in LCQ due to treatment (points)	4.8 (2.2–6.2)	1.8 (0.2–3.6)	4.9 (3.1–6.5)	0.0153
Initial VAS (mm)	69.0 (43.5–80.0)	57.5 (40.0–80.0)	69.0 (49.0–80.0)	0.4729
Change in VAS due to treatment (mm)	38.0 (27.0–56.0)	8.0 (− 15.0 to 35.0)	41.0 (27.0–58.0)	0.0188
Initial capsaicin threshold C2 (μmol/L)	5.9 (2.0–15.7)	1.5 (1.0–6.4)	7.8 (3.4–15.7)	0.0338
Initial capsaicin threshold C5 (μmol/L)	7.8 (3.9–15.7)	5.9 (2.4–19.5)	7.8 (3.9–15.7)	0.4994
Final capsaicin threshold C2 (μmol/L)	3.9 (2.0–15.7)	1.0 (0.5–1.0)	5.9 (3.9–15.7)	0.0073
Final capsaicin threshold C5 (μmol/L)	7.8 (3.9–15.7)	1.0 (1.0–3.9)	11.7 (3.9–15.7)	0.0219
Change in the capsaicin threshold C2 after treatment (μmol/L)^a^	0 (− 1.5 to 3.9)	− 0.5 (− 6.9 to 0)	0 (0–3.9)	0.1248
Change in the capsaicin threshold C5 after treatment (μmol/L)^a^	0 (0–7.8)	− 6.8 (− 30.2 to 0)	0 (0–11.8)	0.0466

Data are presented as median and interquartile range or numbers and percentages. Statistical analysis included Mann–Whitney U or χ^2^ test

*CVA* cough variant asthma, *BMI* body mass index, *PC*_*20*_ provocative concentration of methacholine causing 20% fall in FEV1, *FeNO* fractional exhaled nitric oxide, *UACS* upper airway cough syndrome, *GER* gastroesophageal reflux, *C2* the lowest capsaicin concentrations of capsaicin evoking two coughs, *C5* the lowest capsaicin concentrations of capsaicin evoking five coughs, *LCQ* Leicester Cough Questionnaire, *VAS* visual analogue scale

^a^Difference between final and baseline C2/C5 threshold

### Treatment effects

As 43/49 (87.8%) patients with CC and BHR responded to anti-asthmatic therapy, the positive predictive value (PPV) of BHR in establishing the diagnosis of CVA was 87.8%. Almost three-quarters of patients (31/43) reported the improvement after ICS + LABA, 10/43 (23.3%) after add-on LTRA and 2/43 patients (4.6%) improved after a short course of OCS. No clinical factors were identified to predict the response after each step of therapy.

### BHR and the response to therapy

No correlation was found between PC_20_ and either change in VAS or LCQ score. There was a weak negative correlation between PC_20_ and body mass index (BMI) (r = − 0.36 *P* = 0.0117) and a significant difference between BMI in patients with marked/moderate BHR (< 1 mg/mL) and mild/borderline BHR (> 1 mg/mL):﻿ 33.2 kg/m^2^ (30.2–35.3) vs. 27.4 kg/m^2^ (24.4–31.4), *P* = 0.0040; respectively.

### Factors related to poor treatment outcome

**Fig. 2:**
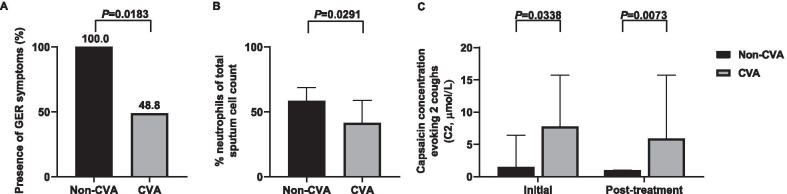
Differences between patients with chronic cough without and with cough variant asthma. Data are presented as median and interquartile range or numbers and percentages. Statistical analysis included Mann–Whitney U or χ^2^ test. *GER* gastroesophageal reflux, *CVA* cough variant asthma, *C2* the lowest capsaicin concentrations of capsaicin evoking two coughs

Patients who did not respond to the therapy, had lower capsaicin threshold (C2) in both, initial and post-treatment capsaicin provocation challenge [1.5 μmol/L (1.0–6.4) vs. 7.8 μmol/L (3.4–15.7), *P* = 0.0338 and 1.0 μmol/L (0.5–1.0) vs. 5.9 μmol/L (3.9–15.7), *P* = 0.0073, respectively], more often reported symptoms of GER (100.0% vs. 48.8%, *P* = 0.0183) and had higher induced sputum neutrophil percentage [62% (49.0–62.0) vs. 41.5% (31.5–51.5), *P* = 0.0291] (Fig. 2).

## Discussion

This study demonstrated high PPV of BHR for the diagnosis of CVA in selected patients with CC suspected to have CVA. Most of these patients showed significant and rapid improvement already after the first-step treatment, i.e., a 4-week course of ICS and LABA. On the other hand, 12% of patients did not respond to treatment despite a high clinical probability of CVA. Low capsaicin threshold in cough challenge, symptoms of GER and high neutrophil percentage in sputum were related to poor response to treatment. The severity of BHR was related to BMI but was ineffective in the prediction of cough reduction after therapy.

To the best of our knowledge, there have been no previous studies on the response to anti-asthmatic therapy in CC adults with BHR. High PPV of BHR in CVA diagnosing in our study suggests that CVA should be considered as a major cause of cough in patients with CC presenting with BHR. Since the diagnosis of asthma is complex, there is no gold standard for its definition [12]. Therefore, there are few previous studies on the diagnostic value of BHR in classic asthma showing diverse PPV of BHR from 0.28 to 0.96 [13–15]. However, it should be mentioned that the assessment was made to diverse asthma diagnostic standards and targeted different groups. To the best of our knowledge, no study assessing the prevalence of CVA in patients with BHR has been published so far. Nevertheless, the analysis of patients with asthma, published by Sistek et al., revealed asthma diagnosis in over 42% with BHR and CC, which was lower than our results [14]. On the contrary, our data showing 87.8% PPV of BHR in CVA seemed to be consistent with data for classic asthma published by Sumino et al., who showed PPV of BHR as high as 96% [15].

Interestingly, the severity of BHR was not the predictor of response to anti-asthmatic therapy. Furthermore, PC_20_ correlated negatively with BMI, which is consistent with previous studies [16] and indicates the significance of obesity in the pathomechanism of CC. As upper airway diseases and gastroesophageal reflux (GER) are both common CC triggers in non-smoking adults and causes of BHR [17], we cannot assume that the presence of BHR in CC is the clear-cut diagnosis of CVA. In this study, all patients with BHR, who did not respond to anti-asthmatic therapy, presented symptoms of GER. It suggests that this population (non-smoking adults with CC, typical GER symptoms and BHR) needs more caution in establishing the diagnosis of CVA.

The results of our study support the opinion that asthmatic cough diagnosis and further decision making on continuation or discontinuation of anti-asthmatic treatment should be based on thorough and objective assessment of response to therapy in patients with suspicion of CVA. This approach is consistent with the recent guidelines [1, 2].

We are aware of several limitations in this study. Firstly, this was a single-center, observational analysis with a limited number of patients. Secondly, the results apply to a highly selected group of adults with CC, without signs of wheezing and dyspnea, with normal spirometry and chest X-ray, in whom CVA was suspected. Thirdly, due to unavailability of the cough monitoring system, we used patient-reported outcomes rather than objective cough measures.

In conclusion, due to its high PPV, BHR may be considered as a reliable predictor of CVA in non-smoking adults with CC.

## Abbreviations

BHR: Bronchial hyperresponsiveness
BMI: Body mass index
CC: Chronic cough
CVA: Cough variant asthma
C2: The lowest capsaicin concentrations of capsaicin evoking two coughs
C5: The lowest capsaicin concentrations of capsaicin evoking five coughs
FeNO: Fractional exhaled nitric oxide
GER: Gastroesophageal reflux
ICS: Inhaled corticosteroids
LABA: Long-acting β_2_-agonists
LCQ: Leicester Cough Questionnaire
ΔLCQ: Change in Leicester Cough Questionnaire from the baseline
LTRA: Leukotriene receptor antagonist
OCS: Oral corticosteroids
PC_20_: Provocative concentration of methacholine causing 20% fall in FEV_1_
PPV: Positive predictive value
QoL: Quality of life
UACS: Upper airway cough syndrome
VAS: Visual analogue scale
ΔVAS: Change in visual analogue scale from the baseline
